# Sprinkler Technology Improves Broiler Production Sustainability: From Stress Alleviation to Water Usage Conservation: A Mini Review

**DOI:** 10.3389/fvets.2020.544814

**Published:** 2020-09-22

**Authors:** Yi Liang, George T. Tabler, Sami Dridi

**Affiliations:** ^1^Department of Poultry Science, University of Arkansas, Fayetteville, AR, United States; ^2^Department of Biological & Agricultural Engineering, University of Arkansas, Fayetteville, AR, United States; ^3^Department of Poultry Science, Mississippi State University, Starkville, MS, United States

**Keywords:** sprinkler, broilers, heat stress, AMPK, HSP, corticosterone, water efficiency

## Abstract

Global poultry production is facing several challenges including a projected increase in global demand for high quality animal proteins and the need to adapt to environmental contrasts including heat stress and the increasing pressure on natural resource (water, land, and energy) availability. Heat stress is one of the most challenging stressor to poultry production because of its strong adverse effects on welfare, production, mortality, and water usage. Most commercial poultry houses worldwide are equipped with a combination of tunnel ventilation and evaporative cooling system (pads, fogging, or low-pressure misting systems) as the status quo to overcome heat stress. Despite prior investments in these systems, critical problems continue to impede poultry production efficiency, which still declines during hot seasons. In fact, these systems tend to saturate the barn air with moisture (>70% relative humidity) which is counterproductive to the bird's own physiological ability to cool itself by hyperventilation (evaporative heat loss). The second challenge with these systems is the significant amount of water usage. This review will summarize some of the benefits of surface wetting of birds through sprinkler technology (SPRINK) that has higher efficiency to maintain birds' comfort with significantly less use of cooling water. Despite higher air temperature and lower relative humidity in the sprinkler house, the SPRINK decreased broiler body core temperature, reduced systemic and intracellular stress, preserved intracellular energy, and averaged six points better FCR compared to evaporative cooling system.

## Introduction

Globally water use is anticipated to increase by 55 percent by 2055 (1), while at the same time surface and groundwater resources for both agriculture use and human consumption will dramatically decrease in the coming decades due to climate change (2). Water is a crucial component for poultry production, not only for bird consumption, but also to alleviate heat stress during the critical cooling periods in tunnel-ventilated broiler houses (3). However, water demand on poultry farms has been on the rise in past years (4). Most of the broiler farms are located in rural area and have limited access to a public water supply, and thus they operate from wells. In addition to the installation and production cost, the well is a direct conduit to the underground aquifer which represent a finite resource because wells may become flow-limited or high-priced to pump due to diminishing groundwater levels (5). Energy consumption and cost of pumping are proportional to volumetric water operation and depth to water table. Cooling system water conservation, therefore, is a worthwhile goal that could reduce peak water demand, costs, energy use, and groundwater depletion.

Animals, including poultry, lose body heat by two mechanisms, i.e., sensible dissipation and evaporation. The sensible heat transfer, including conduction, convection and radiation, is governed by the temperature gradient between an animal and its surroundings. Evaporative heat transfer is governed by the vapor pressure gradient between wet surfaces and its surroundings. The air temperature at which the evaporative heat transfer begins to rise above minimal evaporative heat flux values is 23°C (74°F) (6). As the ambient temperature comes close to the body temperature, evaporative heat flux becomes a major pathway for animals to lose heat to maintain a constant body temperature (7). The partitioning of sensible vs. latent heat loss approximated 40:60 for market age broilers at prevalent summer conditions. During heat stress exposure, body core temperature increases and chickens reduce their energy (feed intake) and divert blood to the periphery (skin) of the body to dissipate heat. This reduction in blood (oxygen) and energy supplies result in stress and damage to many internal organs. The strong adverse effects of heat stress on poultry production, welfare, meat quality, and mortalities are well-documented and elegantly reviewed elsewhere (8–16).

## Mechanical Ventilation and Evaporative Cooling

Air temperature (T), relative humidity (RH), and air velocity (AV) are the main environmental factors affecting homeostasis and performance of broiler chickens. Maintaining birds comfortable and cooling them during hot and humid climate is critical for improving growth performance (body weight gains, feed efficiency, and livability). Improved growth rates and obtaining heavier average market weights contribute to significant heat loads in modern broiler houses. Current strategies used by the poultry industry to alleviate heat stress include tunnel ventilation that uses exhaust fans to speedly move air along the barn length. Higher air velocity increases convective heat loss (wind chill), therefore improving body weight gain and feed conversion efficiency (17–19). Total heat production of market-age broilers increased linearly with air speed in the range of 1.8 m/s (350 fpm) and 2.7 m/s (525 fpm) at high ambient temperature. When wind blow alone no longer delivers sufficient bird cooling, water is circulated over the cooling pads (PAD) to cool the air entering the barn. As a result, the air temperature is decreased by water evaporation, absorbing heat from the air. Unfortunately, high humidity (>70% RH) coupled with hot conditions is counterproductive to the physiological potential of the bird to dissipate heat by evaporation of water from the moist lining of the respiratory tract (20).

A second challenge with recirculating cool cell systems is the significant water usage. The amount of water that a given evaporative cooling pad will use is dependent upon three factors: the amount of air being drawn through the pads, outside temperature and outside humidity. The drier the air is, the more water evaporates into the inlet air, the more cooling the wet pads produce (larger temperature reduction and humidity increase), and the more overall cool cell water use. Newly constructed or renovated broiler houses, which deliver 3.6 m/s (700 fpm) air speed to provide benefits of convective cooling, inadvertently consume a large quantity of cooling water, reaching 280 gallons or more per hour as ambient temperature rises from 29°C (85°F) in the morning to 37°C (98°F) in midafternoon on a typical summer day (5).

## Sprinkler for Surface Wetting

Surface wetting for direct cooling is achieved by sprinkling the surface of livestock or poultry with coarse water droplets so that evaporation occurs locally on the animals (21–23). In a previous laboratory study, Mutaf et al. (24) evaluated the efficiency of cooling laying hens in cages by intermittently sprinkling water onto the head and appendages of hens, and showed a significant stress relief from heat load. Webb and King (25) reported that thermal resistance of plumage of chickens was approximately halved when feathers were wet.

In commercial tunnel-ventilated broiler houses, surface wetting of birds is accomplished using low pressure (280–350 kPa or 40–50 psi), overhead sprinklers (SPRINK), and intermittently operated to apply controlled volumes of coarse water droplets onto the birds (26, 27). On the other hand, foggers or misting nozzles require water line pressure of at least 1,050 kPa (or 150 psi) achieved by supplemental pumps to decrease droplet size for better vaporization (28, 29). The direct cooling of birds and bypassing treating the air in the house by SPRINK permitted major benefits on productivity and litter quality over conventional PAD or fogger systems.

In comparison with a PAD system during a five-flock study over three summers in two commercial-contracted broiler houses, the surface-wetting cooling achieved six-point better feed conversion ratio, although the bird live weight and livability were not significantly different between SPRINK and PAD (27). The range of cooling water application rates of 0.1–0.2 L/day-bird (approximate to 0.17–0.33 mL/min-bird) for birds older than 35 days may be translated into latent heat loss rate of 7–14 W per bird. The total heat production of modern broilers (Cobb × Cobb males) under thermoneutrality was reported to be 7.6 W/kg (30), or 19 W per bird at 2.5 kg. Hence, depending on the thermal condition, evaporation of applied surface water would be responsible for more than half heat dissipation of the bird as sensible heat loss diminishes in heat stress condition.

### Sprinkler Reduces Relative Humidity in the Broiler Houses

Sprinkler cooling requires drier environment for faster evaporation from wetted chicken surface and litter in broiler condition, due to larger vapor pressure gradient between wet surfaces and its surroundings. By wetting the chicken surface without humidifying the air, inside temperature under SPRINK was close to or slightly lower than the ambient temperature, significantly warmer with up to 20% lower relative humidity than a PAD house in comparison (27). This is beneficial because hot air is known to have higher capacity to absorb moisture (water vapor) before reaching saturation (a property called higher vapor pressure deficit). Adequate airflow through the SPRINK house removes moisture in the micro-environment of the chickens. On the contrary, air of 27°C (80°F) and 80% relative humidity in PAD systems does not give much chance for the moisture from drinker leaks or manure to evaporate, increasing the potential of wet litter. Wet litter, in turn, threatens animal welfare, flock health, and performance.

Using five summer flocks between 2009 and 2011 in field-setting trials, Liang et al. (27) have shown that the dew point temperatures in the SPRINK houses were below those of the PAD houses, indicating that air inside the SPRINK houses was drier during supplemental cooling periods. Furthermore, the average litter moisture contents in SPRINK houses at the end of the growout cycles were not significantly different from those in the PAD houses (27). Although litter inevitably received water from the overhead SPRINK systems, data indicated that the litter moisture content was not an issue.

### Sprinkler Improves Water Usage Efficiency

**Figure 1:**
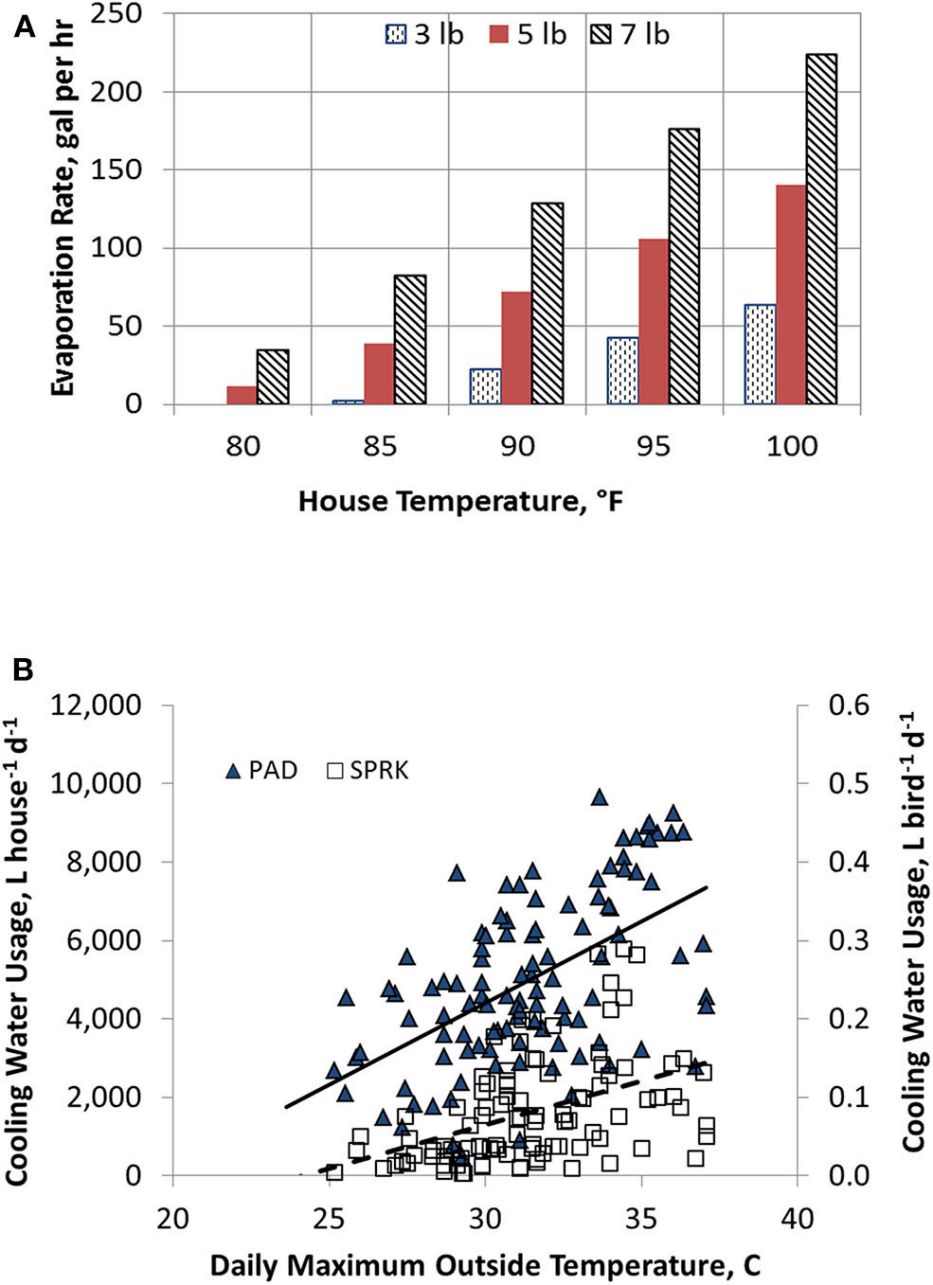
Evaporative rate and cooling water usage in broiler houses. **(A)** Sprinkler water rates increase positively with house temperature and weight of birds in a 40 × 400 ft poultry house. **(B)** Daily cooling water usage as a function of ambient temperatures in sprinkler (SPRINK) and evaporative cooling (PAD) houses. Adapted from Liang et al. (27). Used with permission.

Surface-wetting cooling systems attempt to cool individual birds, not the environment where birds live. Understanding a surface-wetting system requires a re-thinking of how cooling properties work. The phase change from liquid to water vapor taking place on the birds' surfaces is much more efficient in dissipating heat than convective heat transfer between chicken bodies (around 40.5°C) and the warm surroundings (above 27°C). Controllers ramp up sprinkling rates as birds grow and air temperature in the house rise to accommodate the increased demands of heat loads (Figure 1A). Water use by PAD is not directly correlated with bird age. Instead, it depends on three factors: the amount of air passing through the pads, outside temperature, and outside humidity. As outside temperature increases over the course of day, relative humidity will decrease and the amount of water evaporating off the pads will increase. In addition, the number of tunnel fans operating will increase as outside temperature rises, leading to large amount of water evaporating from a PAD system.

Sprinkler cooling consumed 66% less water than that used in a conventional evaporative cooling system during five flocks over three summers in Arkansas (Figure 1B) (27), suggesting a promising technique to conserve water in area with limited water supply. Similar lower water consumption with SPRINK than PAD were demonstrated in Mississippi (unpublished data). As national cooling water usage by growing broilers is estimated to be 825 million gallons/day, SPRINK technology could save 544.5 million gallons/day.

### Sprinkler Improves Broiler Physiological Parameters

**Figure 2:**
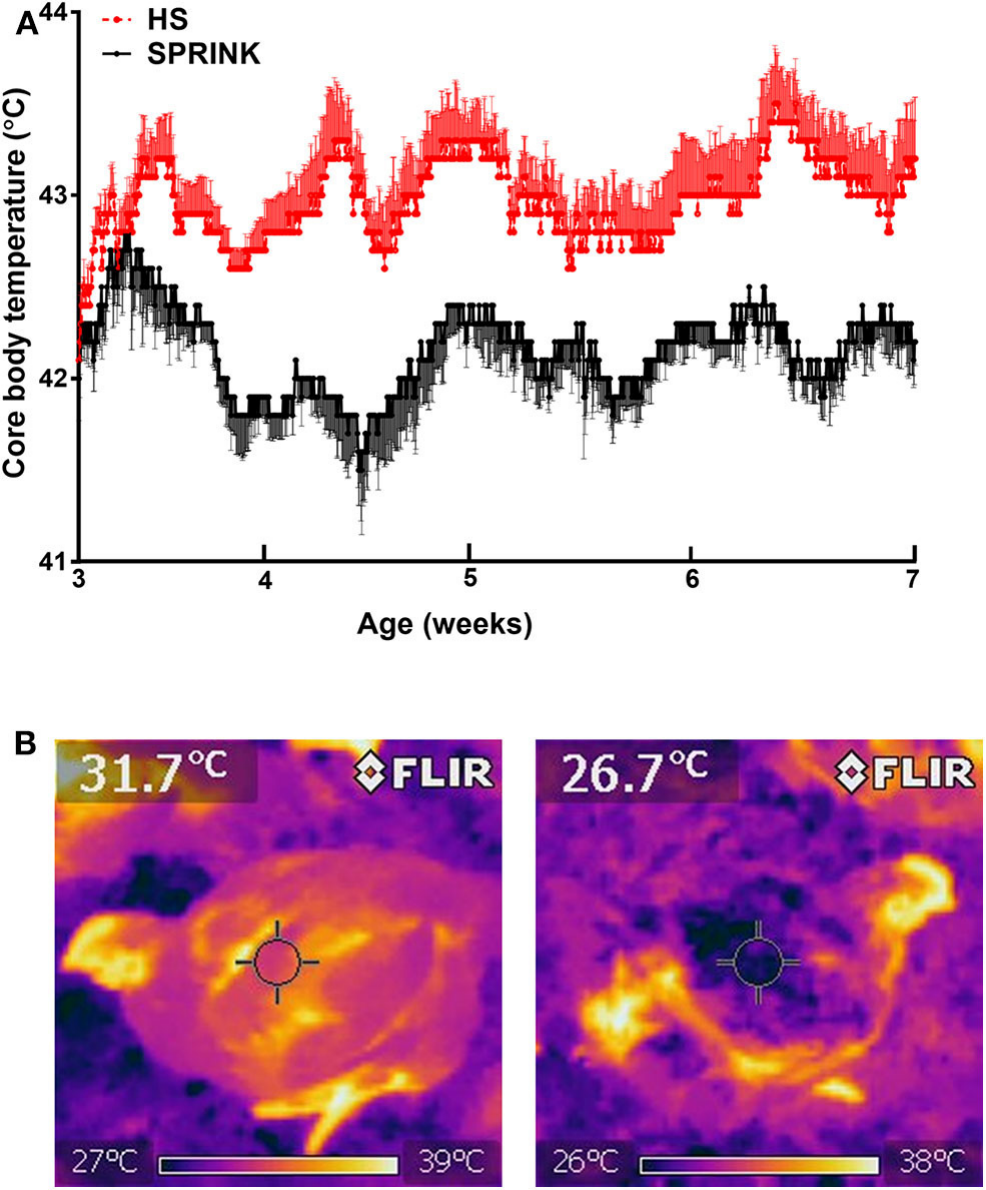
SPRINK reduces core body temperature **(A)** and surface temperature **(B)** in heat-stressed broilers.

**Figure 3:**
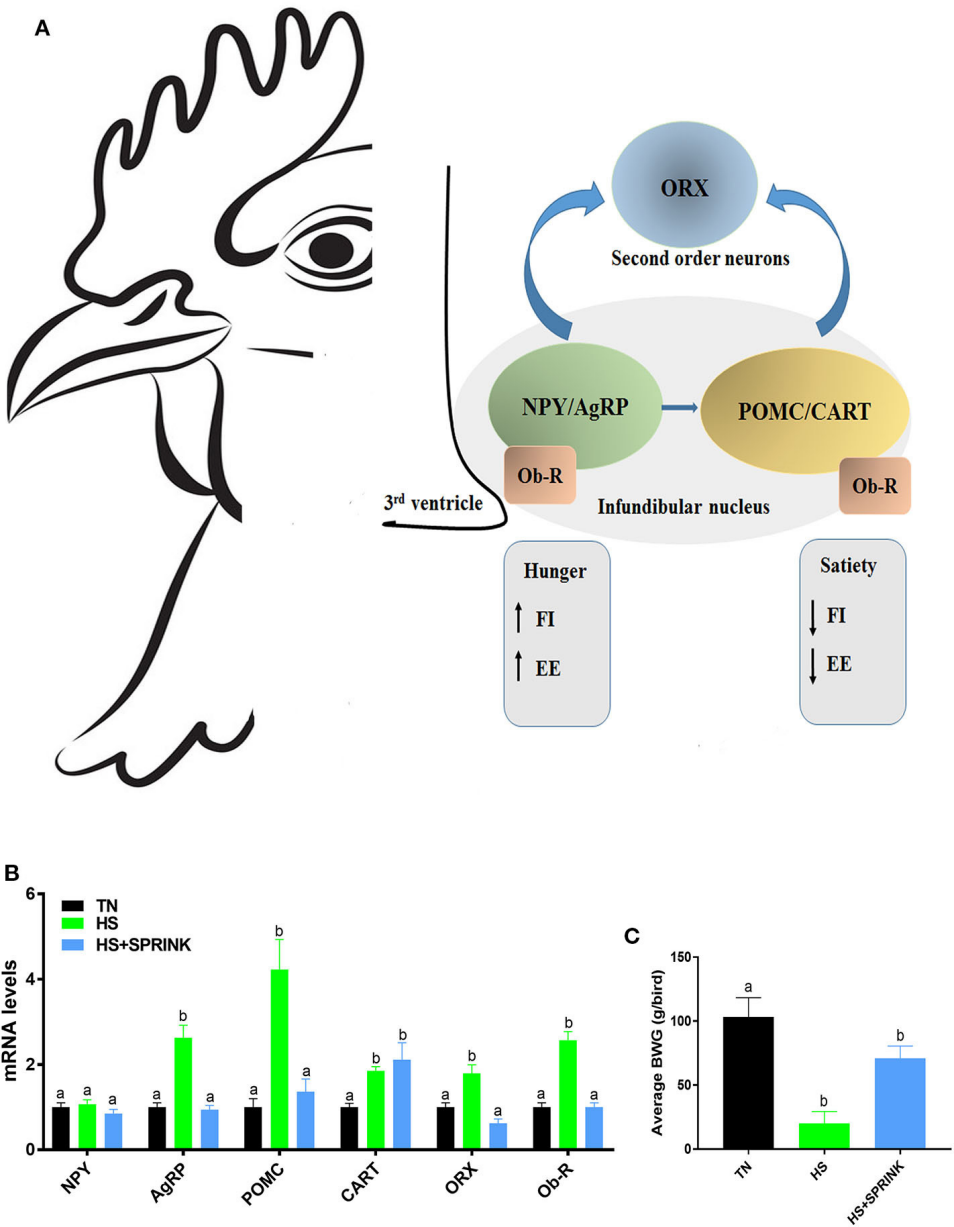
SPRINK modulates the hypothalamic expression of feeding-related neuropeptides **(A,B)**, and improves growth performance [BWG, **(C)**] in heat-stressed broilers reduces core body temperature **(A)** and surface temperature **(B)** in heat-stressed broilers. AgRP, agouti-related peptide; CART, cocaine- and amphetamine-related transcript; EE, energy expenditure; FI, feed intake; HS, heat stress; NPY, neuropeptide Y; Ob-R, leptin receptor; ORX, orexin; POMC, proopiomelanocortin. Figures 3B,C were adapted and modified from Rajaei-Sharifabadi et al. (31) and used with permission.

Mutaf et al. (24) reported significant reduction in body core temperatures (~0.3°C), head (~5.5°C), and dorsal (~3.2°C) surface temperatures of laying hens that received sprinkling compared to those that did not, under thermal stress conditions ranging from 31.3 to 36.0°C. Liang et al. (27) reported insignificant difference of body core temperature monitored continuously between birds raised under either SPRINK or PAD cooled houses over 48 h. In a recent study using broiler chickens, Dridi's group showed that the SPRINK technology reduced body core temperature induced by heat stress by ~0.5–1°C (Figure 2A) (31). Similarly, the SPRINK lowered the overall surface temperature than those with dry surface (Figure 2B). Higher surface temperature indicated vasodilation, which provided an increased temperature gradient between surface and the surrounding air (19). The SPRINK ameliorates feeding and drinking behaviors in heat-stressed broilers, which in turn resulted in better growth performances (body weight gain, BWG) (31). At molecular levels, the SPRINK improves feeding behavior via modulation of the hypothalamic expression of agouti-related peptide (AgRP), proopiomelanocortin (POMC), orexin and its related receptors, and leptin receptor, without affecting cocaine-and amphetamine-regulated transcript (CART) and neuropeptide Y (NPY) expression (31) (Figure 3).

### Sprinkler Reduces Systematic and Intracellular Stress in Broilers

One of the most prominent effects of heat stress is the increased levels of plasma corticosterone, which is considered the gold standard stress marker (14, 32). The SPRINK technology has been shown to prevent the increase of corticosterone levels under heat stress conditions (31). Similarly, Rajaei-Sharifabadi et al. (31) demonstrated that the SPRINK reduces the hypothalamic expression of heat-shock proteins (HSP60 and HSP70) at both the mRNA and protein levels and their transcription factors heat shock factor 1 and 4 (HSF1 and HSF4). Heat shock proteins, a large family of evolutionarily conserved molecular chaperones, play pivotal roles in development and cell survival. They have been shown to confer thermo-tolerance in all organisms being studied (33), but also bestow protection from stressors and insults such as hypoxia and cytotoxic exposure. Initially, they were discovered and characterized as a group of proteins induced in heat-stressed *Drosophila melanogaster* (34, 35), and are now understood to execute critical functions both in stressed and “unstressed” conditions. Stress results in activation of heat shock factor monomers that move from cytosol to nucleus where it combines with other monomers to form a trimer. Trimer of HSFs attaches to promotor site on heat shock gene and induces HSP transcription and translation (36).

### Sprinkler Preserves Intracellular Energy in Broilers

**Figure 4:**
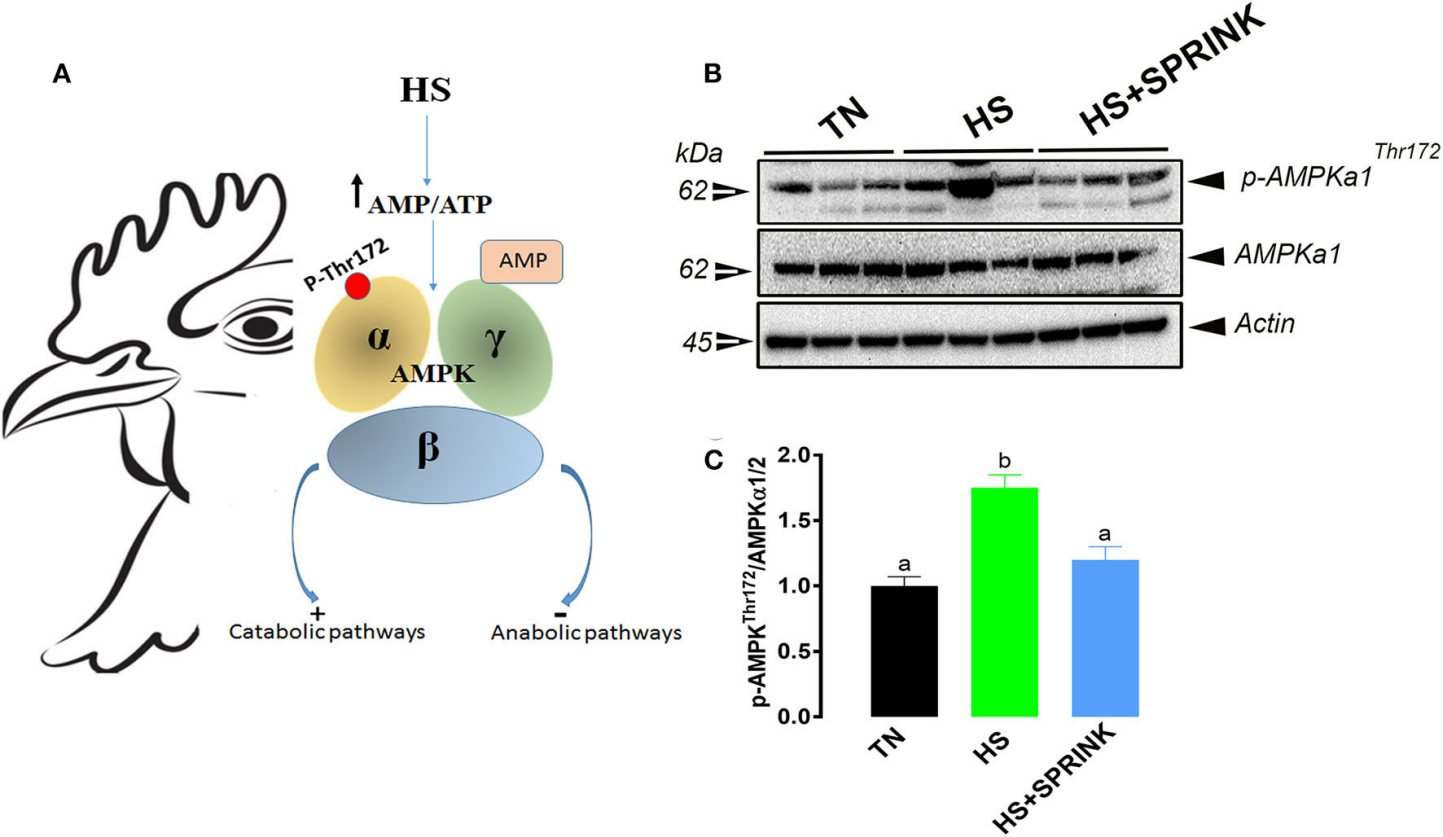
Heat stress reduces the intracellular ATP levels and activates AMPK. AMPK is a heterotrimeric kinase containing a catalytic subunit alpha (AMPKa), and two regulatory subunits beta and gamma (AMPKb and AMPKg). AMPK is activated by AMP nucleotide binding and through reversible phosphorylation on Thr 172 residue **(A)**. Activation of AMPK promotes catabolic pathways to generate more ATP, and inhibits anabolic pathways. SPRINK reverse the effects of HS on AMPK activation and preserves the intracellular ATP levels **(B,C)**. AMP, adenosine monophosphate; AMPK, AMP-activated protein kinase; ATP, adenosine triphosphate; HS, heat stress; TN, thermoneutral. Figure 4B was adapted from Rajaei-Sharifabadi et al. (31) and used with permission.

During heat stress exposure and in order to dissipate body heat, chickens divert blood from internal organs to the periphery (skin) (37). In combination with depressed feed intake, this insufficient blood supply results in low O_2_ and nutrient delivery, hypoxia-like status, and depleted intracellular adenosine triphosphate (ATP) in internal organs. One of the central regulators of cellular and organismal metabolism in eukaryotes is the AMP-activated protein kinase (AMPK), which is activated when intracellular ATP levels drop. AMPK is a highly conserved and master energy sensor that promotes catabolic pathways to generate more ATP, and inhibits anabolic pathways (38, 39). AMPK exists as a heterotrimer, containing a catalytic subunit alpha, and two regulatory subunits beta and gamma (40) (Figure 4A). Under lowered intracellular ATP levels, AMPK can be activated by a direct nucleotide binding of AMP or ADP to the gamma regulatory subunit which in turn leads to a conformational change that protects the activating phosphorylation of AMPK (41) (Figure 4A). Several groups have shown that phosphorylation of Thr172 in the activation loop of AMPK is required for its activation and liver kinase B1 (LKB1) has been shown to mediate this events (42). Hawley et al. (43) showed that AMPK can also be phosphorylated on Thr172 in response to calcium flux, independently of LKB1, via CAMKK2 (CAMKKβ) kinase.

Dridi's group has shown that heat stress increased the phosphorylated levels of hypothalamic AMPKα1/2 at Thr172 site (Figures 4B,C), indicating a depleted levels of intracellular ATP (31). The SPRINK technology prevents AMPK activation-induced by heat stress, suggesting that the SPRINK preserves the intracellular levels of ATP under heat load conditions (31).

## Conclusion and Perspectives

**Figure 5:**
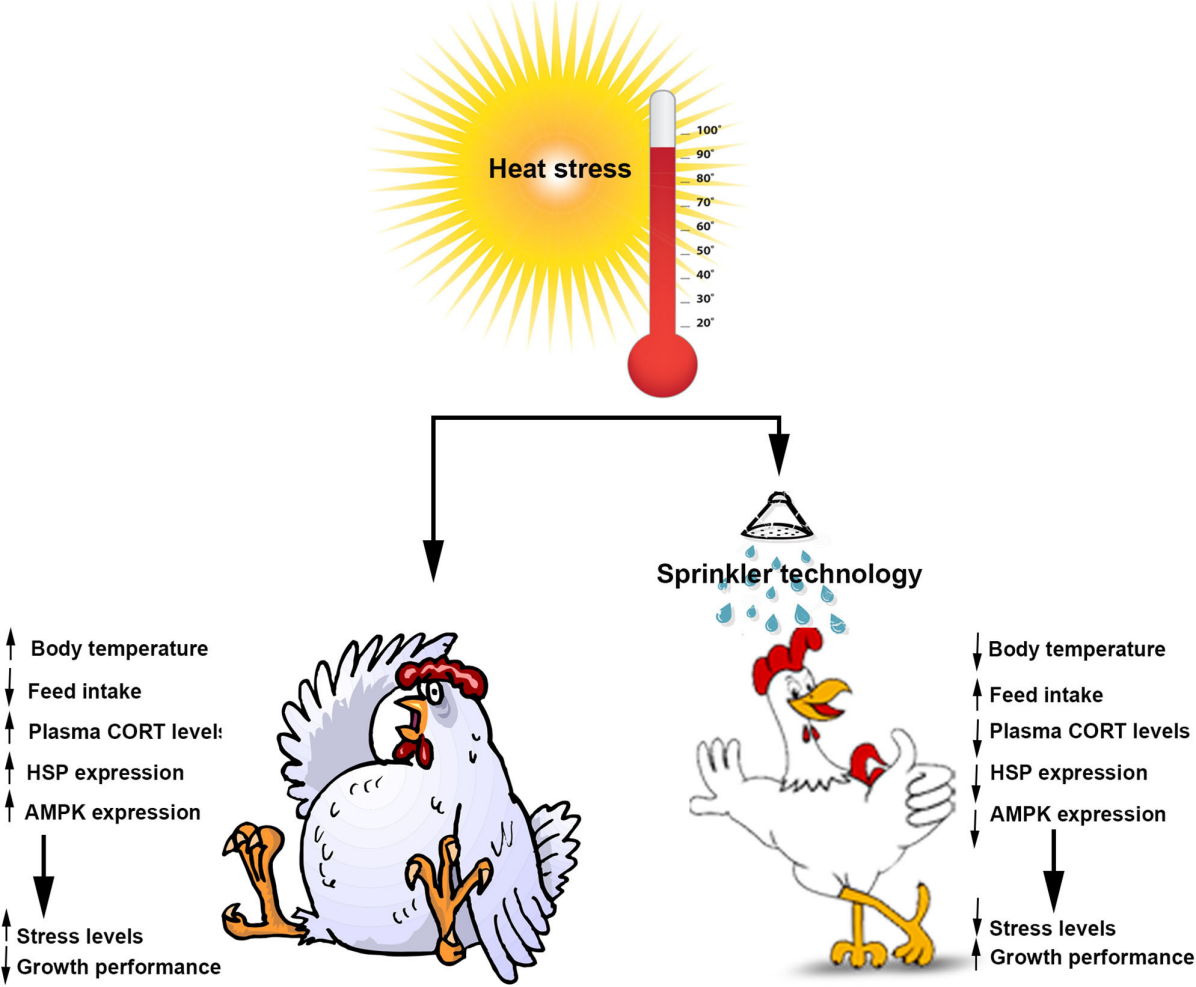
Schematic representation of the beneficial effects of SPRINK in heat-stressed broilers. AMPK, AMP-activated protein kinase; CORT, corticosterone; HSP, heat-shock proteins.

Heat stress is devastating to livestock in general and to the poultry production sustainability in particular, with strong adverse effects on productivity, bird well-being, and mortality associated with heavy economic losses to the industry [for review see (44–47)]. There is, therefore, a critical need for both applied and fundamental researches to identify effective strategies (managerial and/or nutritional) to ameliorate heat stress productivity losses. In addition to 66% water usage conservation, the SPRINK seems to have beneficial effects in preserving the central cellular energy status and mitigating systemic and intracellular stress-induced by heat load, which thereby result in improvement of poultry well-being and growth performance (Figure 5). Further investigations including additional metabolically important tissues such as muscle, liver and gut and high throughput analyses are warranted to further our understanding of heat stress/SPRINK responses.

## Author Contributions

SD wrote the paper with a critical review by GT and YL. All authors contributed to the article and approved the submitted version.

## Conflict of Interest

The authors declare that the research was conducted in the absence of any commercial or financial relationships that could be construed as a potential conflict of interest.
